# A construction waste landfill dataset of two districts in Beijing, China from high resolution satellite images

**DOI:** 10.1038/s41597-024-03240-0

**Published:** 2024-04-16

**Authors:** Shaofu Lin, Lei Huang, Xiliang Liu, Guihong Chen, Zhe Fu

**Affiliations:** 1 Faculty of Information Technology, Beijing University of Technology, Chaoyang District, Beijing, 100124 China; 2 Beijing Big Data Centre, Chaoyang District, Beijing, 100101 China; 3 Administrative Examination and Approval Bureau of the Beijing Economic-Technological Development Area, Beijing, 100176 China

**Keywords:** Environmental impact, Research data

## Abstract

Construction waste is unavoidable in the process of urban development, causing serious environmental pollution. Accurate assessment of municipal construction waste generation requires building construction waste identification models using deep learning technology. However, this process requires high-quality public datasets for model training and validation. This study utilizes Google Earth and GF-2 images as the data source to construct a specific dataset of construction waste landfills in the Changping and Daxing districts of Beijing, China. This dataset contains 3,653 samples of the original image areas and provides mask-labeled images in the semantic segmentation domains. Each pixel within a construction waste landfill is classified into 4 categories of the image areas, including background area, vacant landfillable area, engineering facility area, and waste dumping area. The dataset contains 237,115,531 pixels of construction waste and 49,724,513 pixels of engineering facilities. The pixel-level semantic segmentation labels are provided to quantify the construction waste yield, which can serve as the basic data for construction waste extraction and yield estimation both for academic and industrial research.

## Background & Summary

China is currently in a stage of rapid urbanization. The demolition of buildings during the urban renewal process generates construction waste, which is an unavoidable part and leads to significant environmental problems. Construction waste differs from household waste in that it contains hazardous materials. Heavy metals, asbestos, organic compounds, and other harmful organic materials pose a threat to the environment, so they cannot be directly dumped^1^. Furthermore, construction activities may cause agricultural land loss, loss of soil, and air pollution^2^. Waste is generated as a result of mismanagement, and there are devastating environmental concerns about this waste which are being faced by all of us^3^. Due to the increasing amount of waste generated and its associated environmental impacts, there is an urgent need to accurately estimate the amount of construction waste so as to measure the cost of the urban renewal process^4^. Currently, the identification methods of construction waste primarily include manual field investigation and remote sensing monitoring. Due to the widespread geographic distribution of construction waste landfills^5^, artificial field investigations must be performed, which require a lot of human and material resources, yielding a low work efficiency. Based on remote sensing data sources, researchers have studied the spatial distribution of garbage dumps and solid waste using machine learning technology. For example, Ramnarayan *et al*. proposed a machine learning augmented approach to quantifying and recycling construction and demolition waste^6^. Lu *et al*. estimated construction waste generation in China’s Greater Bay Area using machine learning^7^ technology. However, traditional artificial field investigation and machine-learning-based approaches suffer from the manual selection of feature variables. These methods are only effective for specific types of wastes and it is challenging for professionals to design features that are highly robust and generalizable.

Deep learning-based approaches promise to capture the expertise of professional image interpreters and apply it to train computer-aided tools to support solid waste management^8^. For example, Gao *et al*. proposed a system for identifying and categorizing construction waste using remote sensing images by unmanned aerial vehicles and a multi-layer deep learning approach^9^. Similarly, Zhao *et al*. proposed a method of construction waste recognition based on change detection and deep learning^10^, while Lu *et al*. proposed an automatic identification method for the composition of construction waste mixtures using semantic segmentation in computer vision^11^. Compared with traditional methods, the semi-automated extraction of construction waste from remote sensing images not only saves manpower and material resources but also has high efficiency and a short information extraction period^12^. Open datasets such as ImageNet^13^ and MS COCO^14^ have greatly facilitated the development of deep learning methods for large-scale applications in the field of target extraction, enabling the extraction of rich feature characteristics from remote sensing images more quickly and accurately. The fusion of remote sensing images and deep learning can quickly and accurately obtain the changes of construction waste in construction waste landfills and can accurately estimate the production of construction waste at the macro level^15,16^. However, this method is extremely dependent on high-quality datasets for model training, validation, and testing. There is a dearth of publicly available datasets for construction waste landfill identification, and it is almost impossible to find a uniformly standardized dataset. This gap hinders research on construction waste landfill identification methods. Therefore, the researchers call for more shared solid waste image datasets to be opened for interested researchers to train and evaluate their algorithmic models^17^.

In the field of aerial remote sensing, solid waste recognition based on deep learning has been implemented in very few studies^18,19^. These studies demonstrate the potential of deep learning to achieve image decoding at different scales for various computer vision tasks, such as target detection and semantic segmentation. However, there is a limited number of studies exploring deep learning-based solid waste recognition in aerial remote sensing. The most similar dataset found publicly available is the landfill dataset from the BigEarthNet repository^20^. However, this dataset mainly consists of generic scenes and contains small, coarse images, which may not be ideal for construction waste recognition.

Currently, the main datasets commonly used for construction waste extraction are the AerialWaste dataset provided by Torres *et al*.^21^ and the SWAD dataset provided by Liming Zhou *et al*.^22^ AerialWaste is an illegal landfill detection dataset containing manually labeled airborne, WorldView-3, and Google Earth imagery. The AerialWaste dataset focuses on illegal landfill detection and includes manually labeled airborne, WorldView-3, and Google Earth imagery. The dataset provides information about the type of solid waste, storage methods, site types, and evidence and severity of violations, making it a valuable resource for detecting illegal landfills. The SWAD dataset, on the other hand, is based on remote sensing images collected from Google Earth in Henan Province, China. It contains 998 extended images from WorldView-2 and SPOT satellites in the format of JPG, covering various scenes, including urban, village, and mountainous areas, and includes different types of solid waste such as gravel, slag, industrial waste, and domestic waste. While AerialWaste and SWAD can be excellent datasets for extracting solid waste from remote sensing images, there are still several drawbacks. Firstly, AerialWaste does not provide the labeled information required for semantic segmentation, resulting in the classification being done only for the presence or absence of solid waste rather than on a pixel-level basis, which hinders the quantitative analysis of waste yield. Secondly, the spatial resolutions of the satellite images used in SWAD datasets are not at a sub-meter level, making it challenging to clearly distinguish construction waste landfills and identify typical features. The lack of detail in the images also makes it difficult to accurately differentiate the shape of the landfill and identify smaller surrounding facilities. Additionally, the low resolution may obscure slight colour differences between the construction waste landfill and its surroundings, and the height and shape of the waste pile may not be clear enough for accurate estimation of landfill activity. Table 1 lists several existing CNN-based waste datasets. It can be seen that solid waste datasets in aerial images can be used for detection, classification, or segmentation.

**Table 1:** Comparison of Waste Datasets for CNN.

Dataset	Image Quantity	Instance Quantity	Category Quantity	Image Size	Annotation Type	Shooting Distance	Data Source
SWAD^22^	1,996	5,562	1	1200 × 600–2400 × 1200	Detection	Long	WV2, SPOT
AerialWaste^42^	10,434	10,434	2	700 × 700–1000 × 1000	Classification	Medium and long	AGEA, WV3, GE
UAVVaste^43^	772	3,716	1	—	Detection	Medium	UAV
MJU Waste^44^	2,475	2,532	1	640 × 480	Segmentation	Short	Camera
CWLD(Ours)^30^	3,653	10,959	5	200 × 300–1800 × 1000	Segmentation	Long	GF-2, Google Earth

Based on these several key shortcomings identified, this paper proposes a new dataset, the Construction Waste Landfill Dataset (CWLD), to meet practical needs. The CWLD is designed to be used for training and evaluating semantic segmentation models, calculating construction waste production, and environmental monitoring and management. The dataset is constructed based on several criteria:

- The images come from different sources and have varying quality.
- The images are classified with the pixel-wise level.
- The images are associated with mask-labeled images that provide detailed information about the internal area of the entire construction waste landfill.
- The annotations are curated by professional photo interpreters who specialize in using remote sensing images for landfill detection.

**Fig. 1:**
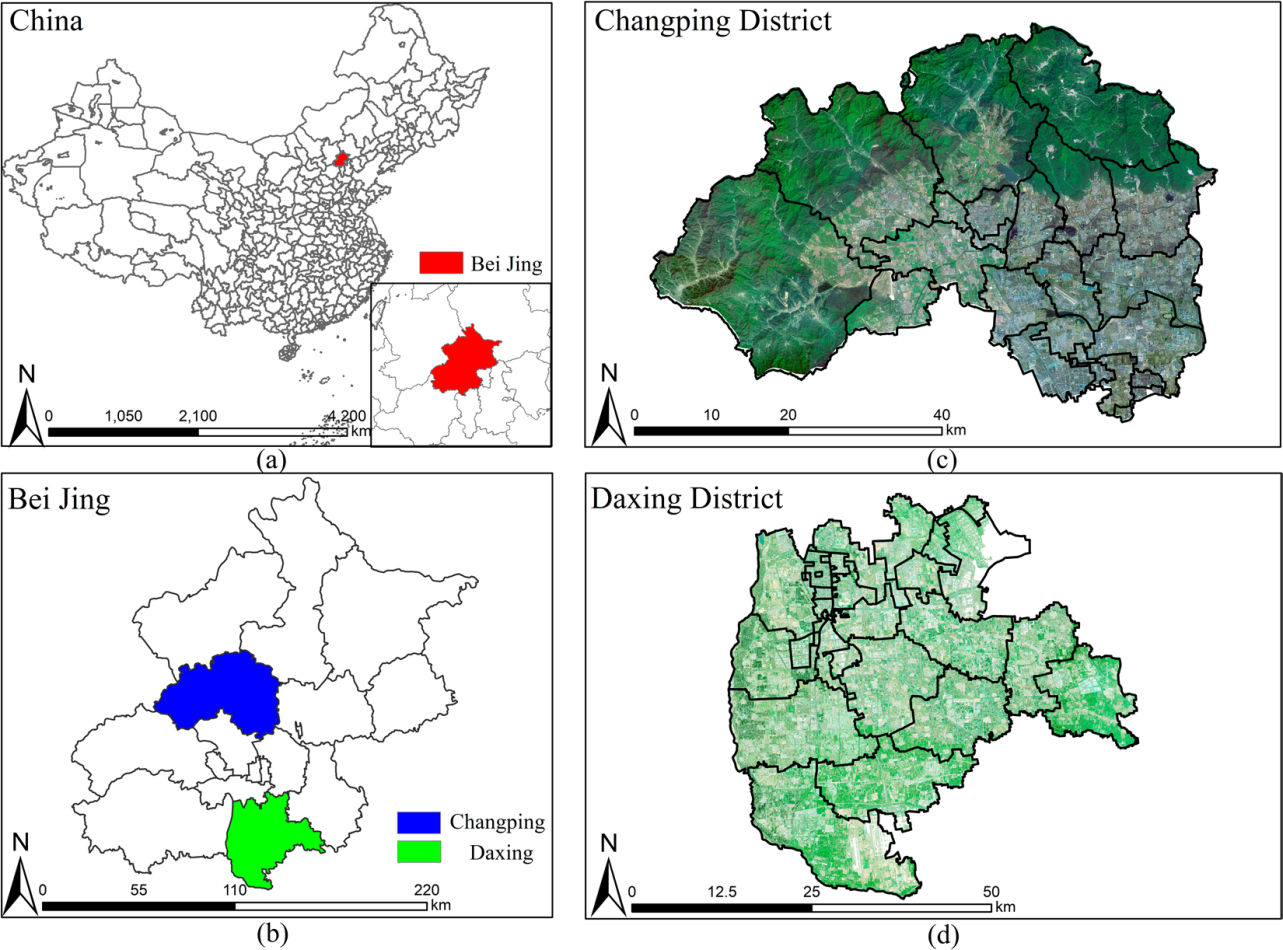
Overview of the study area: (**a**) Beijing’s location in China, (**b**) the location of Changping and Daxing districts in Beijing, where blue colour indicates the Changping district and green colour indicate the Daxing district, and (**c**,**d**) are the raw remote sensing images of the study area.

The CWLD dataset consists of construction waste landfills in Changping and Daxing districts of Beijing, China (as shown in Fig. 1), and uses the Google Earth and GF-2 satellite remote sensing images as the data source. It includes 3,653 samples of original image regions with mask-labeled images for semantic segmentation. The dataset contains 237,115,531 pixels of construction waste regions and 49,724,513 pixels of engineering facilities regions. Providing semantic segmentation labels at the pixel level allows for the quantification of construction waste production, which can provide basic data for the study of high-resolution remote sensing images of construction waste yield and extraction.

## Methods

CWLD selects two representative study areas in Changping and Daxing districts of Beijing, as shown in Fig. 1, for data collection. To enhance the authenticity and credibility of the dataset, we incorporated the spatial coordinates into high-resolution remote sensing images of the study area. By utilizing the officially published geographic coordinates of the construction waste landfill stations, we conducted manual screening and cutting to identify the locations of construction waste landfill sites on the remote sensing images where spatial coordinate information was available.

### Data sources

In this study, raw images are downloaded from the Google Earth and GF-2 remote sensing satellite.

- Images of Changping District are downloaded from the GF-2 satellite. The GF-2 satellite was launched in February 2014 and is the second high-resolution optical Earth observation satellite developed by the China National Space Administration (CNSA) as part of the China High-Resolution Earth Observation System (CHEOS). It incorporates all-digital technology and is equipped with a 0.8-m panchromatic camera and a 3.2-m multispectral camera. Spatial resolution is ≈80 cm GSD. The GF-2 satellite enables all-weather, all-day, high-resolution observation and detection, providing high-quality and clear image information. As a result, it has been widely utilized in remote sensing research^23–27^.
- Images of Daxing District are downloaded from Google Earth using the Google API. Spatial resolution is ≈50 cm GSD. The size of the images is ≈200 × 300–1000 × 1000 pixels. Google images are free to the public and have been used in different remote sensing studies. Their use must respect the Google Earth terms and conditions^28^.

**Fig. 2:**
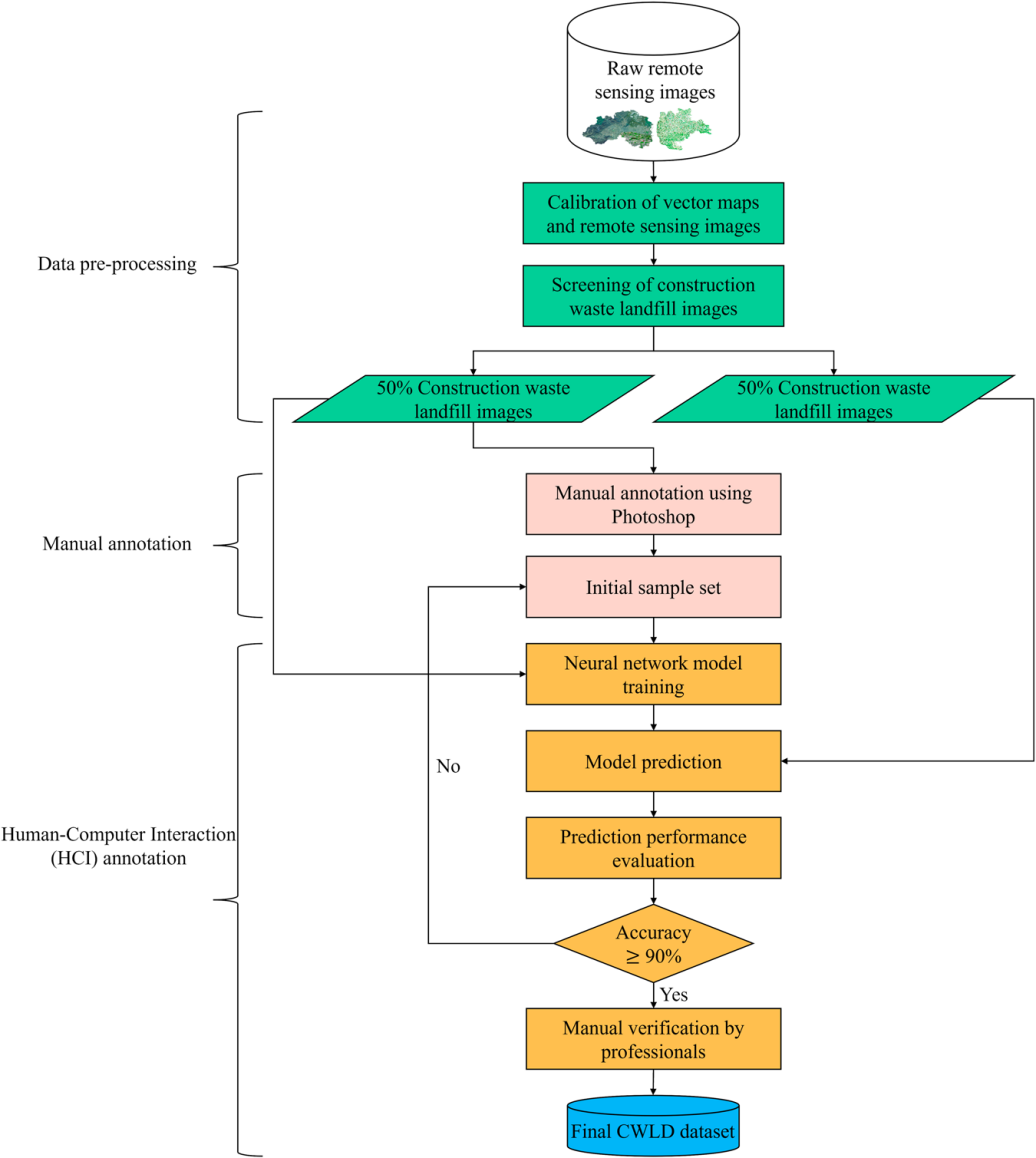
The data annotation process.

Two representative study areas in Changping and Daxing districts of Beijing were selected as the target areas for data collection, with specific information provided in Table 2. The process of creating the CWLD dataset is illustrated in Fig. 2.

**Table 2:** Information about the study areas.

Region name	Latitude and longitude ranges	Image time series	Image sizes
Changping District, Beijing	115°50′17″–116°29′49″E, 40°2′18″–40°23′13″N	2019~2020	500 × 500 px
Daxing District, Beijing	116°13′–116°43′E, 39°26′–39°51′N	2016~2021	200 × 300~1800 × 1000 px

In the data preprocessing stage, the original satellite images were aligned, cut, and filtered to isolate the landfill images. During the manual annotation stage, data annotation software such as Photoshop was utilized to annotate 50% of the landfill images, forming an initial sample set. Subsequently, a neural network model was trained using this initial sample set to equip the model with initial processing capabilities. In the human-computer interaction annotation stage, the trained model was employed to label the remaining 50% of the data interactively. Finally, the dataset production was completed by obtaining segmented labeled images for all the data.

### Data set creation

**Fig. 3:**
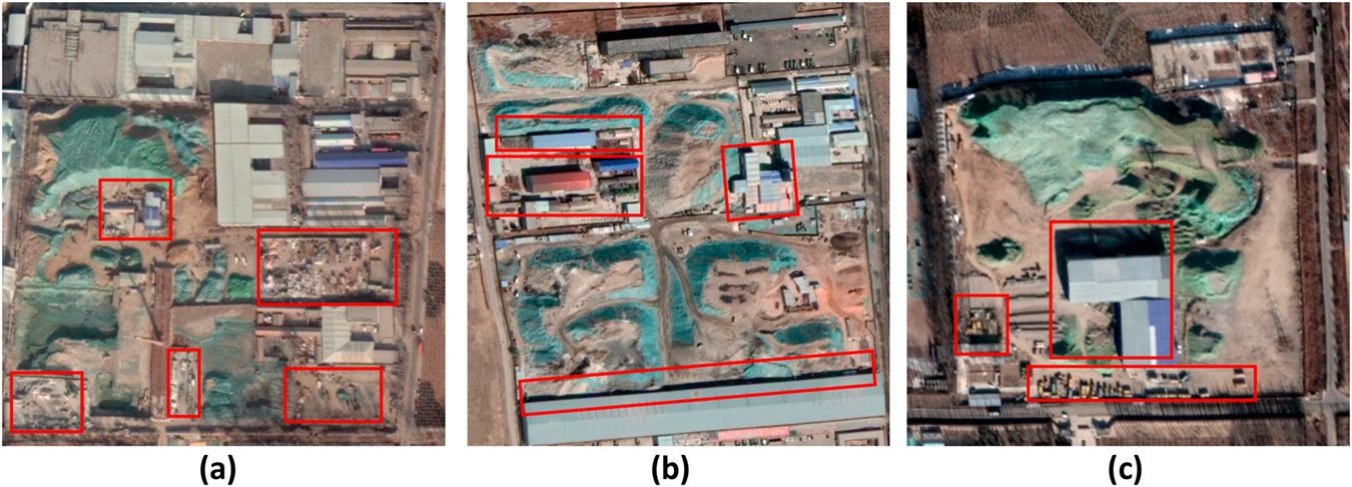
Examples of construction waste landfills. All images within the dataset exhibit significant characteristics of construction waste landfills. As can be seen from the red box, (**a**) The presence of diverse types of solid waste, including plastics, metals, and wood. (**b**) Overlapping of construction waste with the boundaries of associated engineering facilities and buildings. (**c**) Shadows cast by buildings partially cover certain areas. These examples (**a**–**c**) clearly demonstrate the complexity of the internal areas within the construction waste landfills depicted in the images. They showcase variations in shapes, sizes, and orientations, all exhibiting accumulations of different materials and dispersed waste.

CWLD provides a comprehensive representation of the intricate construction waste landfill scenes captured in remote sensing images, offering detailed segmentation for each landfill’s interior. Analysis of satellite imagery reveals that construction waste landfills exhibit a diverse array of shapes, sizes, and orientations, lacking uniformity in their internal areas. Typically, these landfills display a cluttered arrangement of buildings, engineering structures, solid waste, and construction debris, often exceeding the boundaries of the designated structures. Additionally, the area may encompass various other types of solid waste, such as plastics, metals, and wood, among others. Figure 3 illustrates an example of a construction waste landfill depicted in remote sensing images.

**Fig. 4:**
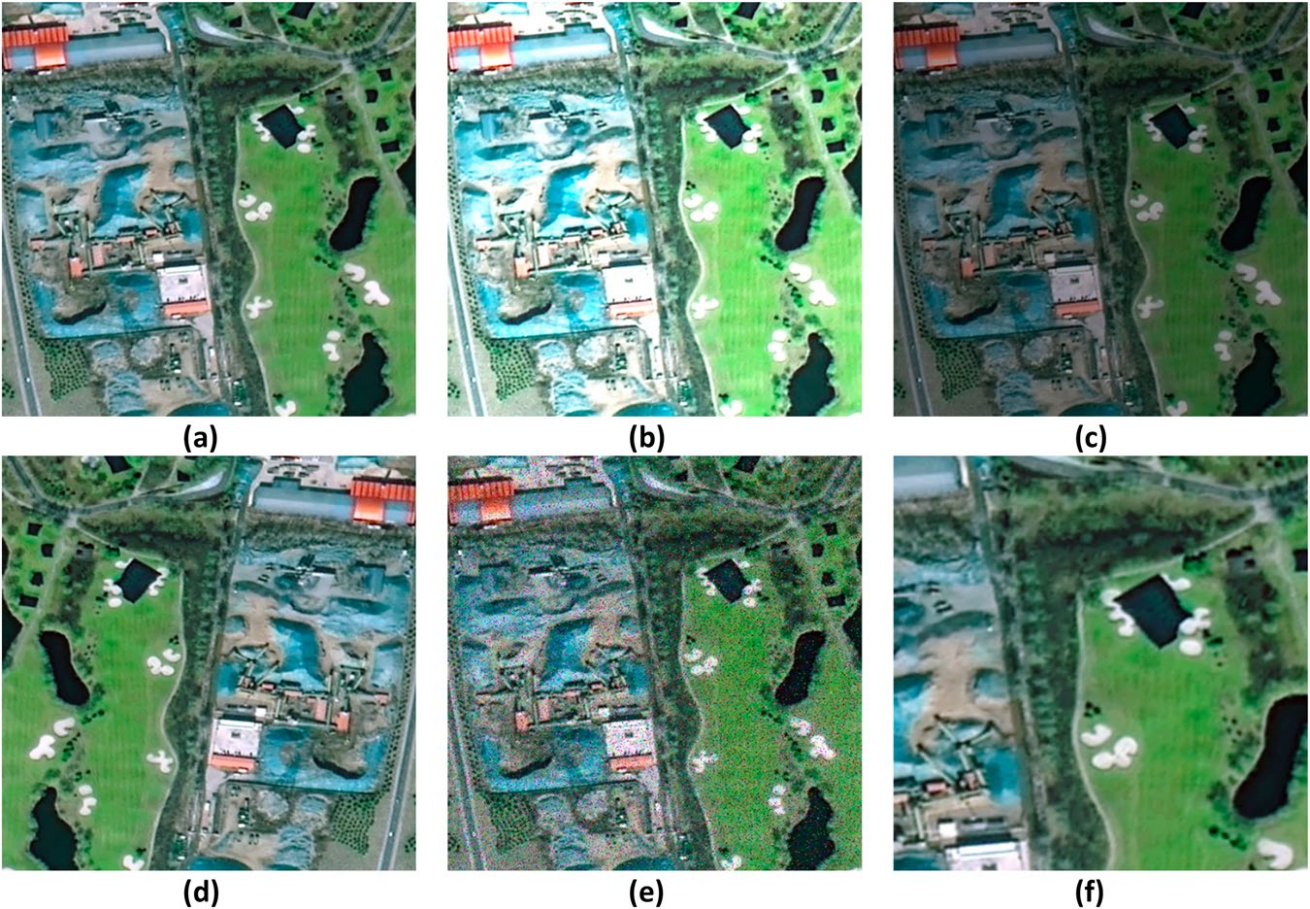
Data enhancement methods. (**a**) Original image: The original image of a construction waste landfill. (**b**) Brightening by 50%: The brightness of the original image is increased by 50%. (**c**) Darkening by 50%: The brightness of the original image is decreased by 50%. (**d**) Mirroring: The original image is flipped horizontally, resulting in a mirrored image. (**e**) Introducing Gaussian noise: A small amount of Gaussian noise is added to the original image, creating a slightly distorted version. (**f**) Random scaling: The original image is randomly scaled up or down, resulting in a larger or smaller version of the image.

Based on Fig. 2, the process of creating the dataset is further described in five main steps:

- Calibration of vector maps and remote sensing images: Utilize ArcMap^29^ software to query and acquire vector maps of Changping and Daxing districts in Beijing, along with their spatial coordinates. Then manually geo-reference the remote sensing images by aligning them with the vector maps using the geographic alignment function, and write the spatial coordinate information and export the aligned images in the format of TIF through ArcMap.
- Images Cutting: Utilize the obtained geographic coordinates of the construction waste landfills in Changping and Daxing districts. Locate the corresponding areas of construction waste landfills in the remote sensing images based on the spatial coordinate information. Cut out 228 images of size 500 × 500 pixels in Changping district and 457 images ranging from 200 × 300 to 1800 × 1000 pixels in Daxing district. The resulting images will all exhibit distinct features of construction waste landfills.
- Define annotation standards: Combine the manually labeled construction waste landfill image set with pixel-level classification features obtained from semantic segmentation. Assign different colours to delineate specific areas within the construction waste landfill, including: (1) Background areas surrounding the landfill are labeled with RGB (0,0,0). (2) Vacant landfillable areas within the landfill are labeled with RGB (255, 255, 255). (3) Buildings and engineering facilities areas within the landfill are labeled with RGB (0, 0, 255). (4) Areas where waste has been deposited within the landfill are labeled with RGB (255, 0, 0).
- Human-computer interaction annotation stage: First, In the manual annotation stage, 50% of the samples are labeled using Photoshop software to form an initial sample set. Subsequently, the neural network model is trained according to the initial sample set so that the model has the ability to segment the construction waste landfill with high accuracy. Finally, in the human-computer interaction annotation stage, the model is used to pre-annotate the remaining 50% of the data, and on the basis of the pre-annotation, professionals manually verify each labeled image to more finely annotate the category of each pixel.
- Data enhancement: Data enhancement techniques are applied to increase the diversity, generalizability, and robustness of the training dataset. Since the number of actual landfills may be limited, data expansion methods are employed, including brightness adjustment (50% brighter and 50% darker), introducing Gaussian noise, mirroring, and random scaling. These techniques are performed on the existing dataset to generate new samples with variations, as shown in Fig. 4.

**Fig. 5:**
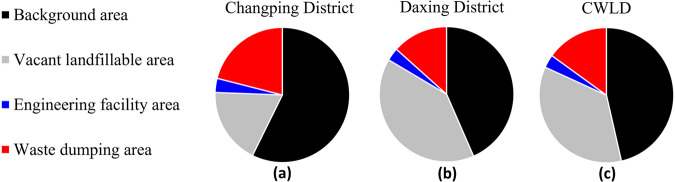
The percentage of each type of label in the dataset, (**a**) Changping District, (**b**) Daxing District, and (**c**) CWLD.

Figure 5 illustrates the proportion of the four types of labels within the construction waste landfill dataset. It is evident that the landfill occupies approximately 53.58% of the dataset. Notably, the proportions of each label type in the Daxing District dataset are consistent with those in the overall construction waste landfill dataset, indicating the high quality of the dataset. These features highlight the heterogeneity of the dataset, which in turn enhances the complexity of landfill identification and waste detection tasks. Moreover, these diverse features can be effectively utilized to train network models.

**Fig. 6:**
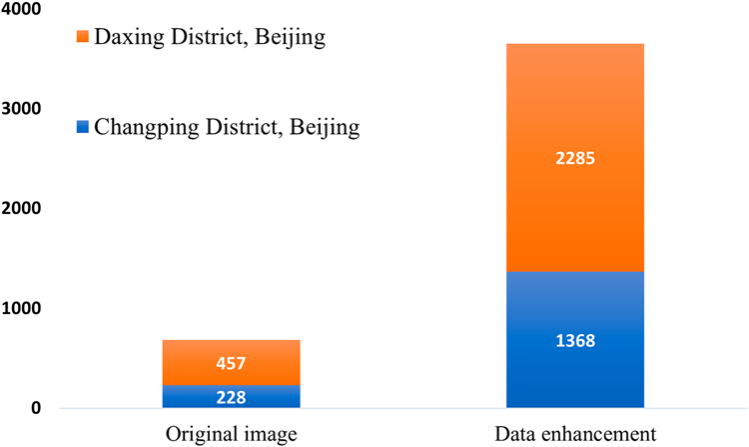
Distribution of data sample resources.

Figure 6 displays the distribution of data from various sources, resulting in a total of 3,653 images depicting construction landfills within the study area.

### Data quality control and utilization processes

**Fig. 7:**
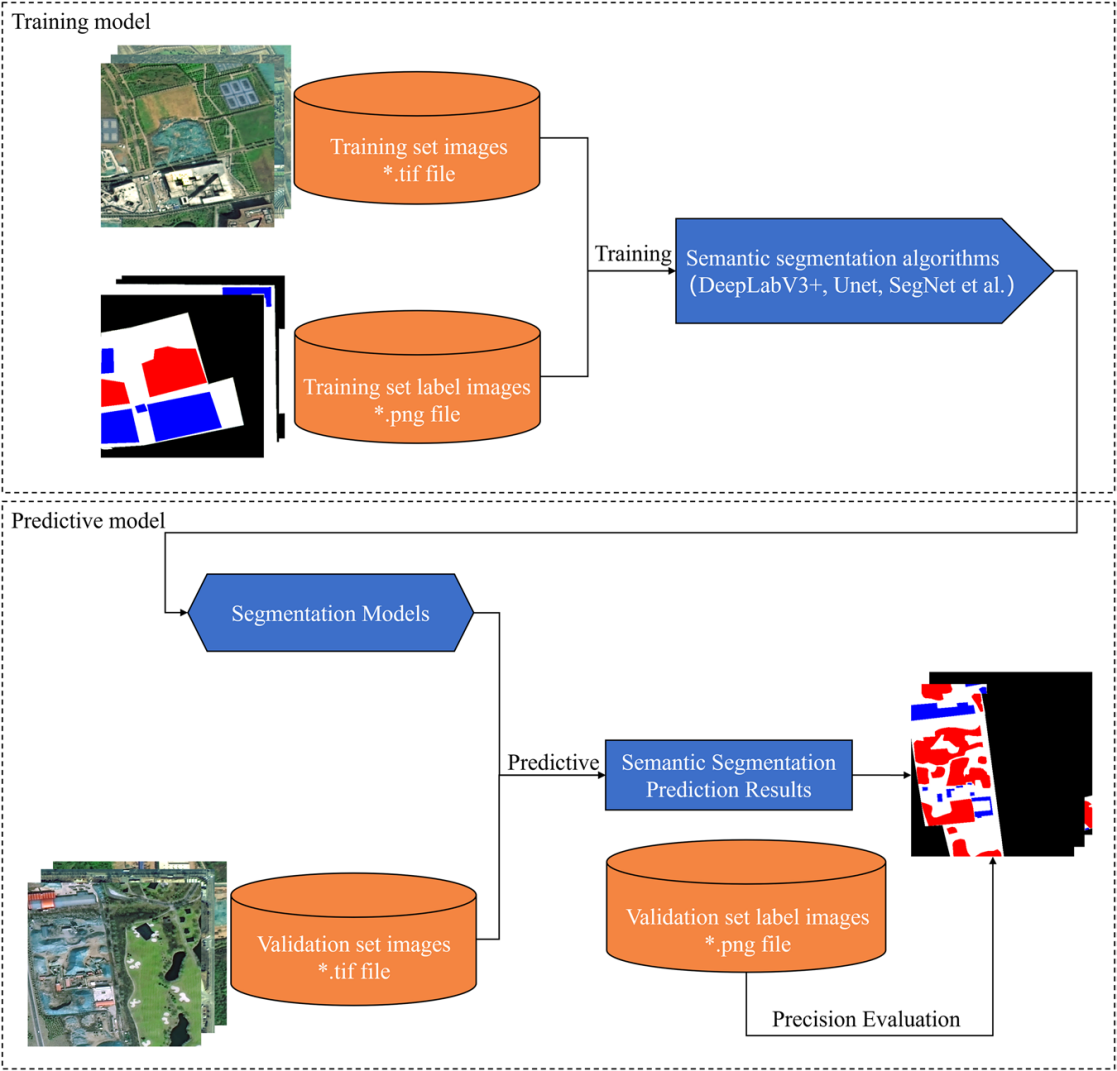
Illustrates the dataset utilization process. The dataset utilization process involves loading the training and test images into a deep learning model (such as DeepLabV3 + , UNet, SegNet) using Python’s PIL library and relevant modules/packages from the OpenCV library. The model is capable of performing pixel-level semantic segmentation tasks. To evaluate the predictive performance of CNN models, standard metrics such as Accuracy, Precision, Recall, and F1-score are utilized for quantitative prediction assessment.

To ensure data quality, a comprehensive quality control process has been implemented throughout the stages of image acquisition, pre-processing, manual labeling, and human-computer interactive labeling. Skilled technicians carry out image acquisition and labeling following standardized operating procedures. Multiple checks are conducted to ensure the reliability, completeness, and uniformity of the labeled data. The pre-processing phase involves cutting the original image batch based on the geographic coordinates of the construction waste landfill site. Secondary screening and manual examination are performed to identify images that exhibit characteristics of the construction waste landfill site. Distorted, blurred, or otherwise problematic images are manually removed to minimize interference factors. Figure 7 provides an illustration of the dataset utilization process.

### Detailed description of the dataset

**Fig. 8:**
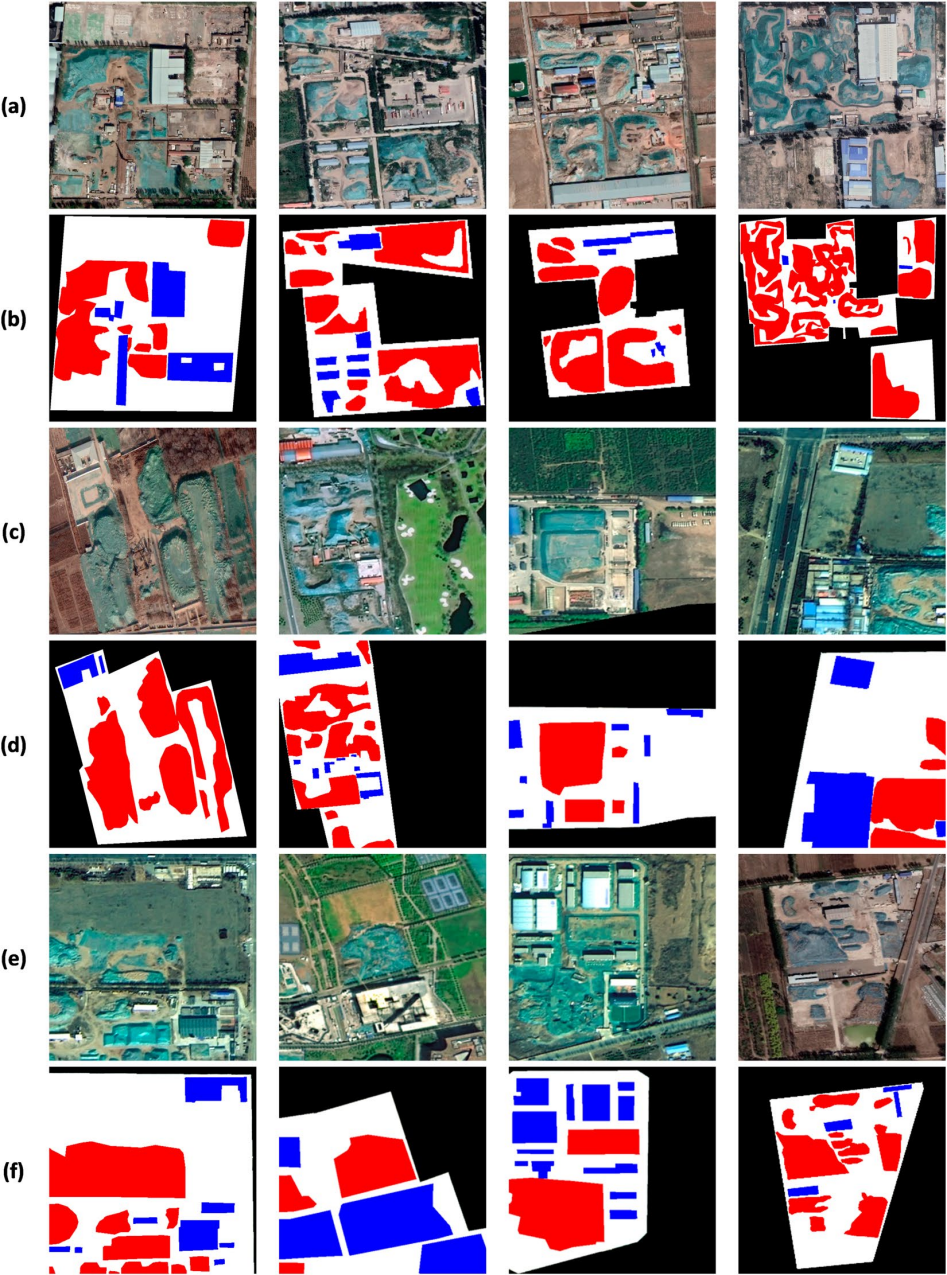
Illustrates the presentation of the sample dataset. Groups (**a**), (**c**), and (**e**) depict remote sensing images, while groups (**b**), (**d**), and (**f**) represent the corresponding mask-labeled images. In the mask-labeled images, the white area represents vacant landfillable, the black area indicates the image background, the blue area represents the engineering facility area, and the red area represents the dumping area.

The CWLD dataset contains 3,653 samples, each of which depicts clear construction waste landfill features. These images contain one to three types of targets. With the high spatial resolution of the dataset, it is easy to distinguish various ground features, thereby the dataset can fulfil the requirements of deep learning network model training tasks, as demonstrated in Fig. 8.

## Data Records

The CWLD dataset is available in the Zenodo repository^30^. The structure of the repository consists of three folders: Original Dataset, Construction Waste Landfill Dataset, and Deep Learning Datasets. The remote sensing images are in *.tif format, and the labeled images are in *.png format. Table 3 provides an overview of the file organization within the dataset, and this organization allows the reader to easily select the desired data.

- **Original Dataset**. This folder contains raw remote sensing images cut directly from Google Earth and GF-2 imagery that have not been processed by data enhancement techniques and the corresponding labeled images. It is stored according to different areas, including 228 images in Changping District and 457 images in Daxing District.
- **The Construction Waste Landfill Dataset**. Due to the limited number of actual construction waste landfills, the sample size is too small to support the training model. Therefore, the raw data are enriched using data enhancement techniques such as brightening, darkening, introducing noise, mirroring, and random scaling, resulting in a total of 7,306 pieces of data, which are stored separately according to the remote sensing images and the labeled images, which are 3,653 pieces each.
- **Deep Learning Datasets**. In order to accommodate the fixed input size requirement of neural network models, the data needs to be pre-processed. In this folder, the input data is adjusted to 512 × 512 pixels, which may result in image stretching and distortion. The folder is further divided into training and validation sets in the ratio of 8:2, where the training set is 2,922, and the validation set is 731. Each subfolder contains remote sensing image data files and segmentation label image files.

**Table 3:** Organization of the dataset files.

Parent directory	Subdirectory	Number of images	Content of the document	Description of the document
Original data	Changping District	228	images/*.tif	Original images
			label/*.png	Original image labels
	Daxing District	457	images/*.tif	Original images
			label/*.png	Original image labels
Construction Waste Landfill Dataset	images	3,653	images/*.tif	CWLD images
	label	3,653	label/*.png	CWLD label
Datasets for Deep Learning (512 × 512px)	train	2,922	images/*.tif	Training set images
			label/*.png	Training set image labels
	val	731	images/*.tif	Validation set images
			label/*.png	Validation set image labels

The dataset can be utilized for pixel-level-based semantic segmentation tasks using Python’s PIL library and related modules or packages from the OpenCV library. These images serve as inputs to the deep learning model, following the same approach as standard datasets used in traditional semantic segmentation tasks.

## Technical Validation

### Quantitative analysis of different models on the dataset

The CWLD dataset has undergone technology validation to evaluate its application in semantic segmentation for building deep learning predictive models that automate the identification and analysis of municipal construction waste landfills. Semantic segmentation is one of the popular research areas in deep learning and image pixel-level classification^31,32^.

To comprehensively analyze the dataset and network performance, six evaluation metrics are utilized, including Accuracy, Precision, Recall, Intersection over Union (IoU), F1 score (F1), and Bayesian error rate (BER). Table 4 specifies that pixels within the waste dumping area are considered positive cases (Positive), while pixels outside of this area are negative cases (Negative). These cases are categorized as True Positive (TP), False Positive (FP), True Negative (TN), and False Negative (FN), forming the confusion matrix. These metrics help evaluate the recognition ability of the model. The definition of the confusion matrix is presented in Table 4.

1. Accuracy: This is one of the most direct evaluation metrics to evaluate the accuracy of the algorithm and refers to the ratio of the number of correctly categorized pixels to the total number of pixels.
  1 $$Acc=\frac{TP+TN}{TP+FP+FN+TN}$$
2. Precision: Precision is the proportion of correct predictions out of the total number of positive predictions.
  2 $$Precision=\frac{TP}{TP+FP}$$
3. Recall: Recall is the proportion of correctly predicted outcomes out of the total number of positive events.
  3 $$Recall=\frac{TP}{TP+FN}$$
4. IoU: IoU is the rate of overlap between predicted and real edges.
  4 $$IoU=\frac{TP}{TP+FP+FN}$$
5. F1: The F1 score is used to balance the relationship between precision and recall.
  5 $$F1=\frac{2\ast Precision\times Recall}{Precision+Recall}$$
6. BER: We introduce Bayesian Error Rate (BER) as a measure of the lowest classification error rate achievable for target extraction, BER is a common evaluation metric in the medical field that indicates the lowest classification error rate achievable for a given model. We compare the Bayesian error with the training set error to determine if it still possesses avoidable bias, if there is room for optimization, and if it is overfitting.

6 $$BER=\frac{1}{2}\ast \left(\frac{FN}{TP+FN}+\frac{FP}{FP+TN}\right)$$

**Table 4:** The confusion matrix of construction waste landfill identification.

Real data	Projected results	
	Waste dumping area	Other areas
Waste dumping area	TP	FN
Other areas	FP	TN

**Fig. 9:**
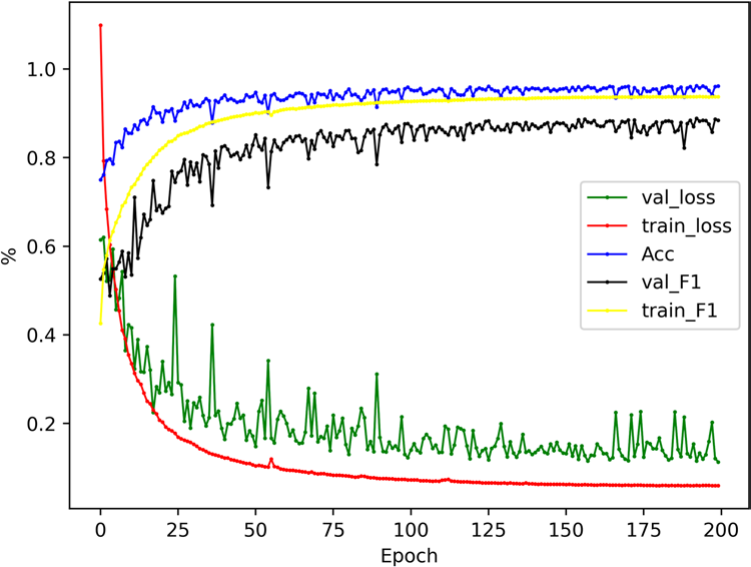
Changes in indicators during model training, where the horizontal coordinate is the number of training rounds and the vertical coordinate is the parameter size. From the curve, it can be clearly found that with the increase of training rounds, the loss value gradually decreases and tends to fit, and the Accuracy and F1 scores continue to rise and finally reach equilibrium.
- Additionally, we evaluated classical networks in the field of semantic segmentation, including UNet^34^, SegNet^35^, and PSPNet^36^. These networks were validated using the dataset, and the results demonstrated that the semantic segmentation model trained from the dataset effectively recognizes various regions within the construction waste landfill. The experimental results are presented in Table 5. Detailed comparative information on the ACC and F1-score metrics for the four networks on the dataset is provided in Fig. 10.

In order to adapt to the complexity of construction waste landfill images, we trained a multi-scale convolutional neural network structure generalized to complex scenes and validated it on a large-scale dataset and the validation process can be summarized as follows:

- The dataset is divided into two parts, 80% of the images are used for training and 20% for validation.
- The construction of the improved DeepLabV3+^33^ network training model can be divided into four main components including the backbone network, the Atrous Spatial Pyramid Pooling (ASPP) module, the encoding structure and the decoding structure.
- The improved DeepLabV3 + network was applied to the training set of CWLD to construct predictive models. The performance of these models was then tested on the validation set. The classifier achieved **96.21% Accuracy, 88.28% Precision, 90.24% Recall, 88.89% F1 score, 82.08% IoU, and 5.07% BER**. Figure 9 illustrates the changes in the metrics during the model training.

**Table 5:** Quantitative comparison of different neural networks on validation sets (%).

Model	Acc	Precision	Recall	F1	IoU	BER
UNet	95.57	88.28	90.15	88.89	82.87	5.16
SegNet	95.72	88.18	90.24	88.89	82.08	5.07
PSPNet	92.69	82.85	79.80	79.55	69.41	10.98
Improved DeepLabV3+	**96.21**	**88.56**	**90.70**	**89.01**	**82.95**	**4.93**

**Fig. 10:**
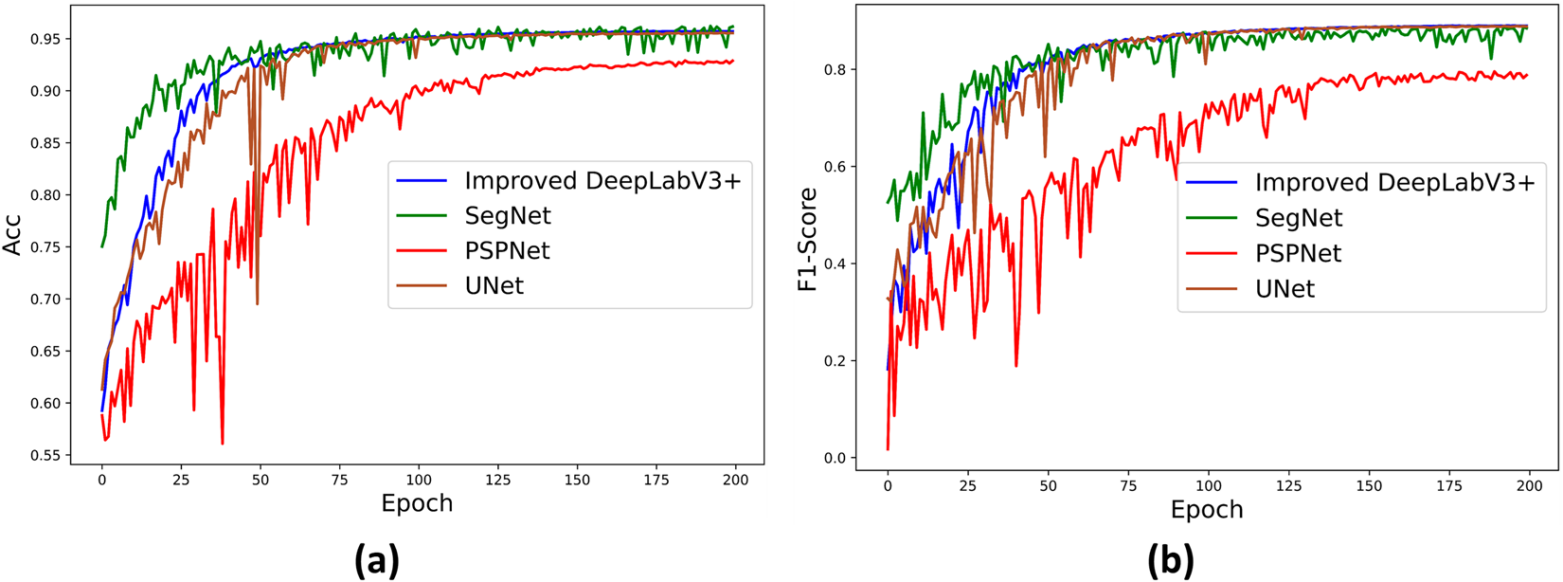
Accuracy and F1-Score curves of different models, (**a**) Accuracy curves; (**b**) F1-Score curves.

From Table 5 and Fig. 10, it is evident that the semantic segmentation model trained on the dataset is highly effective. In comparison with UNet, SegNet, and PSPNet, the Improved DeepLabV3 + network model achieves better segmentation results in terms of accuracy (Acc) and F1-Score. Specifically, when compared to the poorer-performing PSPNet network, the Improved DeepLabV3 + network model delivers 3.52% higher accuracy and 9.46% higher F1-Score in segmentation.

### Classification network architecture

**Fig. 11:**
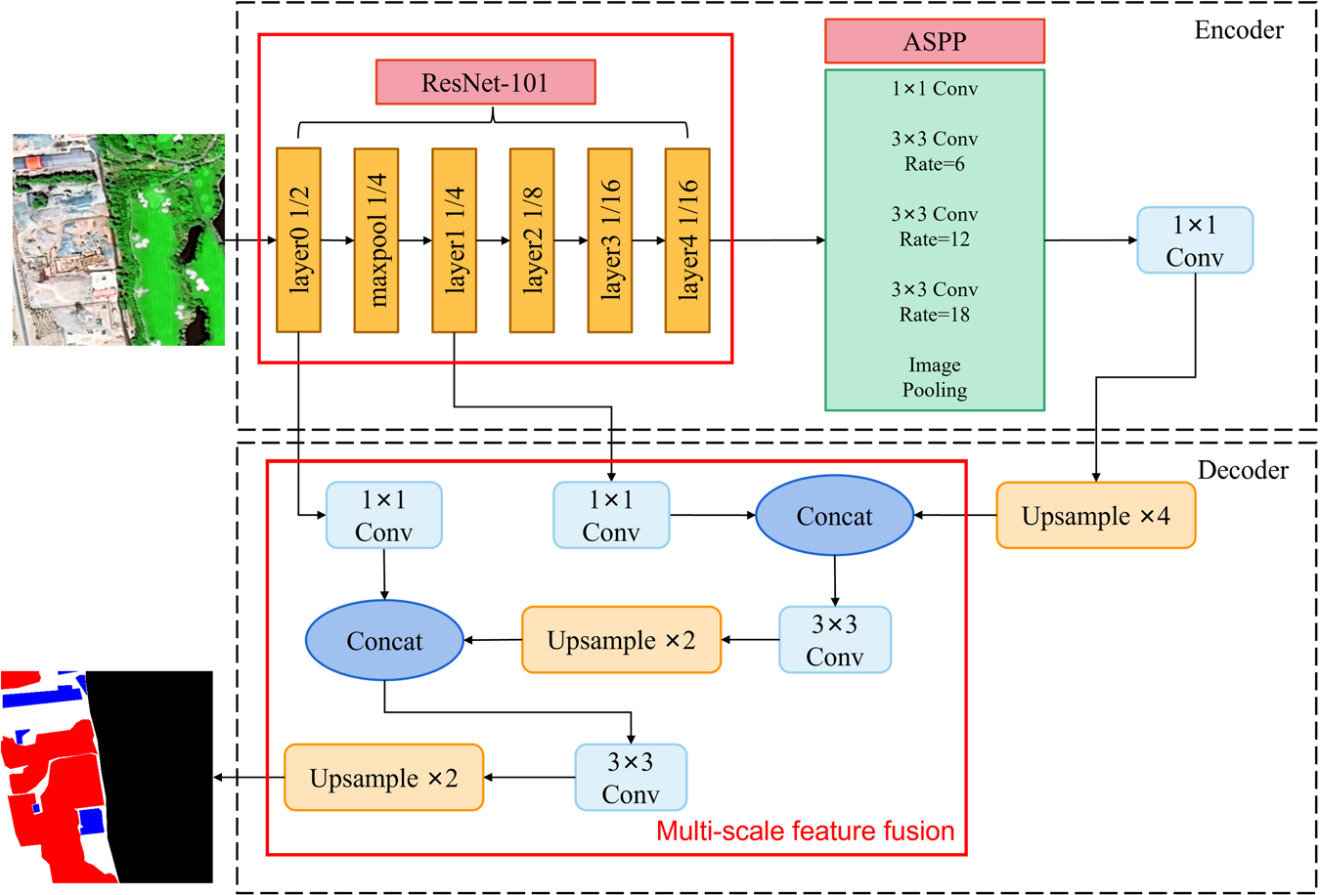
Improved DeepLabV3 + network using ResNet-101 and feature fusion techniques for technology validation in CWLD.

The DeepLabV3 + network is a multi-scale, multipath parallel convolutional neural network proposed by Chen L.C *et al*. in 2018, known for its excellent image segmentation performance and robustness. Its core innovation lies in the encoding-decoding structure that combines low-level semantic information with high-level semantic information, thereby improving the segmentation accuracy of the network. In this paper, we present an improved version of the DeepLabV3 + network. As shown in Fig. 11, the network is divided into four main components, including the backbone network, the Atrous Spatial Pyramid Pooling (ASPP) module, the encoding structure, and the decoding structure.

**Fig. 12:**
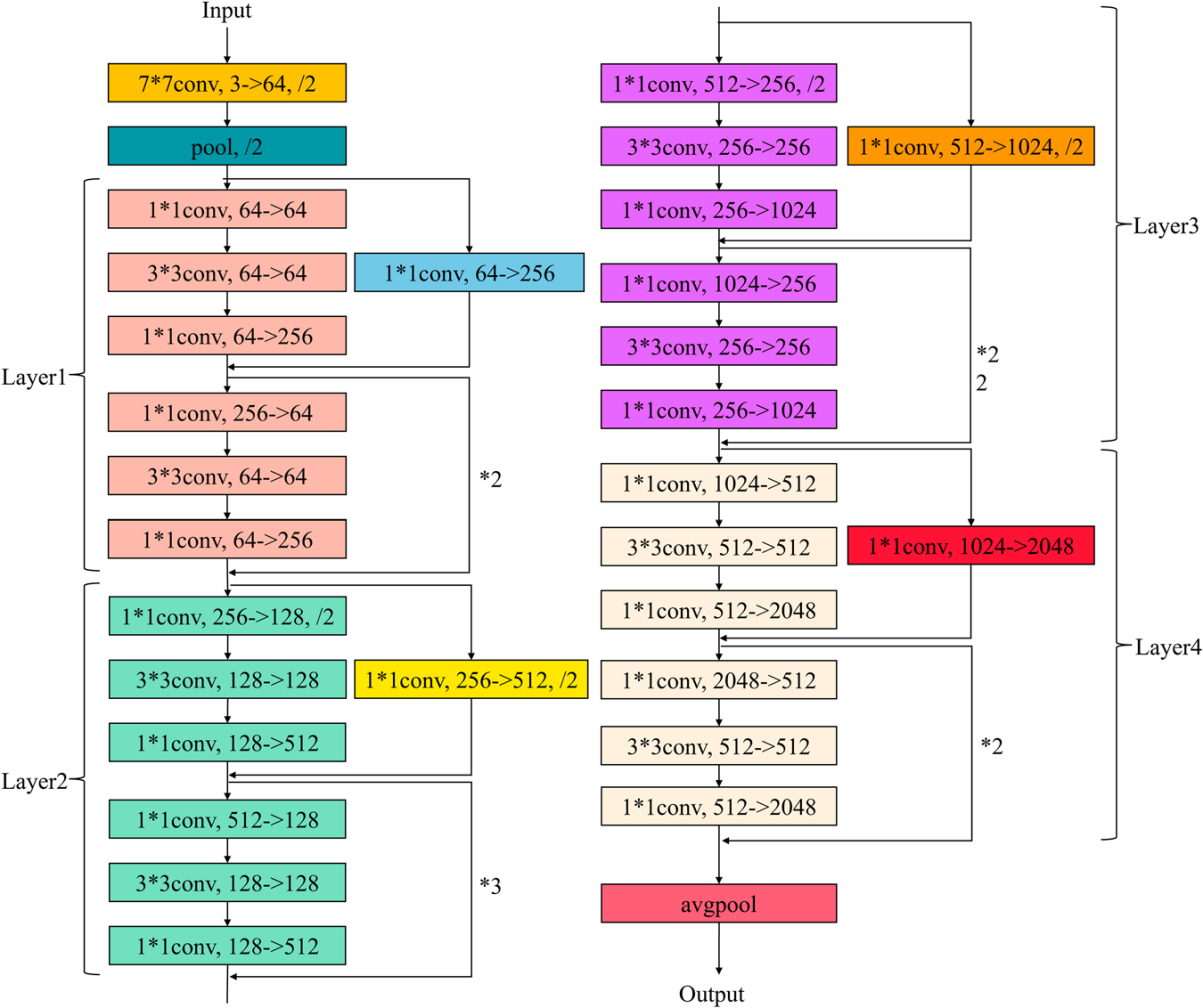
ResNet-101 network structure.

The backbone network of the improved DeepLabV3 + model utilizes ResNet-101, a deep convolutional neural network in the ResNet series^37^. ResNet-101 consists of four convolutional groups and a pooling layer, which helps extract features from the original image. The structure of the ResNet-101 network is shown in Fig. 12.

Compared to shallower network structures like ResNet-34 and ResNet-50, ResNet-101 has a deeper network structure, allowing it to capture image features more effectively and improve model performance. With more layers and convolutional kernels, ResNet-101 can better learn image features, enhance model accuracy, and exhibit improved generalization ability.

The Atrous Spatial Pyramid Pooling (ASPP) module, another component of the improved DeepLabV3 + network, employs Atrous convolutions with different expansion rates to fuse features at various scales. This expansion increases the receptive field without sacrificing image information.

The encoding structure is responsible for feature extraction while reducing the size of the feature map, thereby reducing computational complexity. On the other hand, the decoding structure performs up-sampling to recover spatial detail information and fuses deep and shallow features to achieve more precise recognition results.

### Qualitative assessment and validation with examples

Quantitative analyses play a crucial role in assessing the performance of a model by providing numerical metrics. These metrics allow for a comprehensive and objective evaluation of the model’s performance, facilitating the optimization of its hyperparameters and enabling comparisons between different models. However, it is equally important to complement these quantitative assessments with qualitative analyses through visual inspection. Such qualitative evaluations help to gain a deeper understanding of the quality of the model’s predictions.

**Fig. 13:**
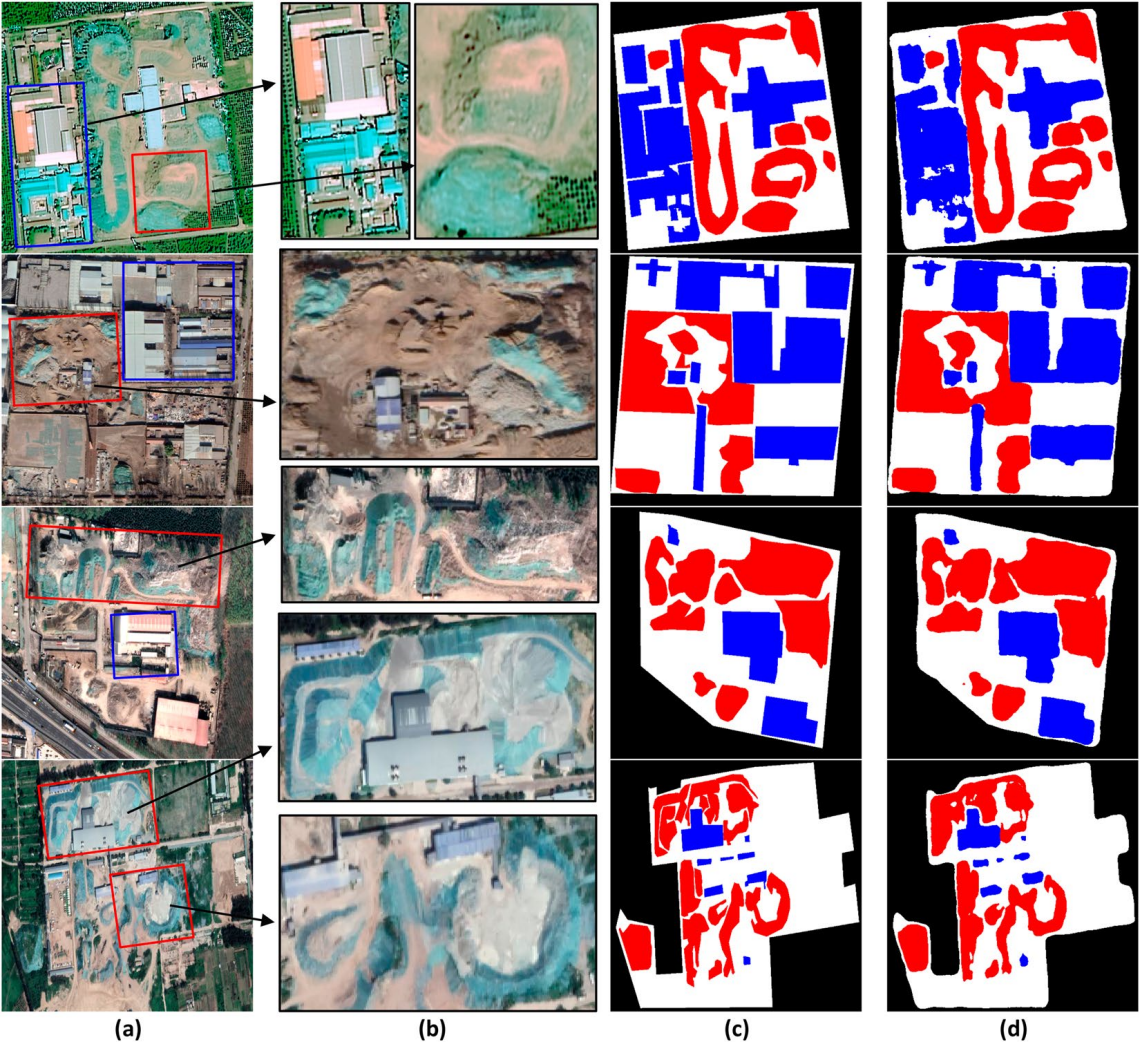
An example of a construction waste landfill projection. In (**a**), the original input sample is overlaid with manually drawn bounding boxes. To provide a closer look at the bounding box region, (**b**) offers a zoomed-in view. The manually mask-labeled image can be seen in (**c**), while (**d**) showcases the image predicted by the enhanced DeepLabV3+ network.

In Fig. 13, we present several examples of correctly predicted images to demonstrate the performance of the model. The first example showcases the model’s ability to effectively distinguish between the construction waste pile area and the vacant landfillable area in an open area model. It accurately recognizes the contours of the waste pile and demonstrates high accuracy in identifying building areas, such as engineering facilities.

The second example features an image with a diverse range of complex features, including small piles of waste, plastics, and other objects. By examining the prediction results, it becomes evident that the trained model’s predictions in this complex scene largely align with the manually labeled image. This indicates the model’s proficiency in handling intricate scenarios.

The third and fourth examples consist of standardized images of a construction waste landfill. In both cases, the model successfully delineates different zones with clear contours between individual construction waste piles. These examples highlight the model’s exceptional segmentation performance and its ability to maintain high accuracy across various complex scenarios.

To summarize, the combination of quantitative analyses and qualitative evaluations through visual inspection provides a comprehensive assessment of the model’s performance. The examples presented in Fig. 13 demonstrate the model’s proficiency in accurately recognizing different areas, contours, and objects, reaffirming its effectiveness in segmentation tasks and its ability to excel in diverse and complex scenarios.

## Usage Notes

### Dataset usage

The dataset described in the paper can be utilized to train models for various tasks, including binary and multi-label semantic segmentation as well as weakly supervised localization. To facilitate access to the dataset, the Code Availability module provides links to public repositories where it can be downloaded. In addition to the dataset itself, the study also presents statistical information about the dataset. This includes details such as the percentage of labels in each category. Distribution plots are employed to visualize this information, allowing readers to quickly grasp the overall characteristics of the dataset. To simplify data loading and batch processing, utilities are provided that leverage the PyTorch DataLoader https://pytorch.org/docs/stable/data.html#torch.utils.data.DatasetPyTorch. This enables efficient handling of the dataset during model training and evaluation.

### Dataset annotation tools

This dataset primarily utilized Adobe Photoshop (PS)^38^ software for labeling.

1. Adobe Photoshop (PS). Adobe Photoshop is a powerful image editing software that can be used for a variety of image processing tasks, including semantic segmentation image annotation.

- **Drawing tool**. Photoshop provides a variety of drawing tools that can be used for different labeling tasks. In semantic segmentation, usually use the Brush tool or the Polygonal Lasso Tool.
- **Labeling method**. The drawing tool is employed to create semantic segmentation labels on a new layer. Different tools can be used to draw lines, polygons, or fill areas to represent various semantic objects in the image. To differentiate between different objects, different colours or labels can be added.
- **Save Label Result**. The labeled image file is saved, typically by saving the label layer along with the original image for future editing or export.

While Photoshop is a powerful tool, it may not be as efficient as specialized image annotation tools. For subsequent labeling tasks, we will use the professional labeling tool, LabelMe.

(2) LabelMe^39^. LabelMe is an online platform and a popular open-source annotation tool used for annotating images and creating labeled datasets for machine learning and computer vision tasks. LabelMe has been widely used in both research and industry for tasks such as object detection, image segmentation, and image classification. It has contributed to the development of labeled datasets used to train and evaluate machine learning models. Key features of LabelMe include:

- **Annotation Variety**. Users can create annotations for object detection, semantic segmentation, and instance segmentation tasks. This versatility makes it suitable for a wide range of computer vision projects.
- **Data Management**. LabelMe facilitates the organization and management of labeled data, making it easy to track annotations and associated metadata.
- **Exporting Annotations**. Annotations can be exported in various formats, including JSON and XML, which are commonly used in machine learning pipelines.

### Supported usage cases and extension

The CWLD dataset is made available to the scientific community to facilitate advances in identifying and accounting for construction waste in remote sensing images and is a public dataset to finely delineate remote sensing images of the interior areas of construction waste landfills.

CWLD can be used to train a multi-class segmentation model for construction waste landfills based on remote sensing images. This model can accurately predict the condition of each area inside a construction waste landfill. From an application point of view, this highly efficient and intelligent identification method is sufficient for relevant organizations seeking to speed up the urban monitoring process. When abnormal changes in the construction waste landfill at a certain location are detected, it can quickly identify the site according to the coordinates and improve the efficiency of urban construction waste management.

Remote sensing images have high spatial resolution, strong timeliness, large amount of information, and macro-observation characteristics. Based on the combination of deep learning and remote sensing data, they can more accurately and quickly detect changes in construction waste in the process of urban regeneration. This is suitable for relevant departments and research institutes to use for preliminary theoretical research and assessment of the development of urban construction waste production measurement products.

### The future extensions of CWLD pursue different directions

- **Geographical expansion**. Currently, the study area is limited to Beijing, which may introduce some selection bias despite considering the diversity of geographic environments and land types. To improve the dataset’s diversity and enable the detection of construction waste landfills in Beijing and other regions of China, there are plans to expand the dataset to include data from other areas.
- **More fine-grained categorization**. The current dataset accurately divides the internal area of the construction waste landfill. However, considering the various types of construction waste and solid waste, it is necessary to identify different types of waste more accurately. To enhance the efficiency of municipal waste management, there are plans to add data on different types of solid waste to the dataset.
- **Multi-modal imagery**. Currently, CWLD only contains RGB images. To increase the dataset’s diversity and support multi-modal data fusion, there are plans to incorporate images captured in different wavelength bands or spectra (e.g., visible, infrared, radar, laser) using various sensors such as satellites, airborne platforms, and ground-based systems.
- **Multi-temporal imagery**. Analysing changes over time in the same area can provide valuable information about landfill activity and facilitate accurate estimation of solid waste production, growth, or decrease^40^. By adding different time series of images of the same site to CWLD, relevant environmental agencies can better plan interventions. There are already numerous cases in the CWLD dataset where different time points have been captured to track changes in solid waste areas.

## Data Availability

The data and predictive models presented in this study are publicly available: **• Dataset**. You can download the images from the Zenodo repository^30^ https://zenodo.org/record/8333888. After downloading, place the train and val files from the Deep Learning Datasets folder into the data folder of the CWLD semantic segmentation model. **• CWLD semantic segmentation model**. Code scripts and project instructions on how to use this dataset to train segmentation models are available for download in the Zenodo repository^41^, and weight files for trained models are also provided for readers to try out the models without training. Visit https://zenodo.org/records/10911443. The requirements.txt file provides the libraries needed to run the project, and the README.md file describes in detail the deployment process and functionality of each module, as well as the role of the various toolkits in utils. In addition, the model and the corresponding code for executing the model are available on the GitHub platform at https://github.com/huangleinxidimejd/CWLD_Model.
